# Evolution of research on global amphibian declines

**DOI:** 10.1111/cobi.70146

**Published:** 2025-09-12

**Authors:** Jordann Crawford‐Ash, Maldwyn John Evans, Tamilie Carvalho, Jodi J. L. Rowley, Trenton W. J. Garner, Erin Muths, Ben C. Scheele

**Affiliations:** ^1^ Fenner School of Environment and Society Australian National University Canberra Australian Capital Territory Australia; ^2^ Department of Ecology and Evolutionary Biology University of Michigan Ann Arbor Michigan USA; ^3^ Institute for Global Change Biology, School for Environment and Sustainability University of Michigan Ann Arbor Michigan USA; ^4^ Centre for Ecosystem Science, School of Biological, Earth and Environmental Sciences UNSW Sydney Sydney New South Wales Australia; ^5^ Australian Museum Research Institute Australian Museum Sydney New South Wales Australia; ^6^ Institute of Zoology Zoological Society of London London UK; ^7^ Unit for Environmental Sciences and Management North‐West University Potchefstroom South Africa; ^8^ Fort Collins Science Center U.S. Geological Survey Fort Collins Colorado USA

**Keywords:** amphibian decline, conservation action, geographic bias, research trends, topic modeling, acción de conservación, declinación de anfibios, modelado de temas, sesgo geográfico, tendencias en la investigació n, 两栖动物数量下降, 研究趋势, 主题建模, 保护行动, 地理偏差

## Abstract

In the late 1980s, the scientific community became aware of severe, enigmatic amphibian population declines. These declines triggered a wave of research focused on quantifying their extent and identifying key drivers. We used text‐analysis techniques, including topic modeling and geoparsing, to examine the evolution of research focused on amphibian declines. We sought to provide an example of scientific inquiry in action, from the initial recognition and quantification of the phenomenon to identifying drivers and understanding mechanisms of amphibian decline. We delineated research topics, tracked spatiotemporal trends from 1985 to 2024, and extracted the number of publications per topic. Early research focused on evaluating the veracity of declines and was followed by investigations of potential drivers (in particular, UVB radiation, pollution, and habitat fragmentation and loss). After the amphibian chytrid fungus (*Batrachochytrium dendrobatidis*) was identified in the late 1990s, research emphasis shifted toward disease. Subsequently, disease‐focused research became increasingly specialized, the primary topics of which were susceptibility, resistance and tolerance, and mitigation. Most recently, extinction risk and climate change became increasingly prominent topics, reflecting emerging threats to amphibians. Regions with high amphibian biodiversity and observed declines (e.g., Central and South America) were underrepresented in the literature, and research was strongly biased toward North America, Australia, and Europe. We uncovered a clear disconnect between the amphibian decline literature and the development of effective management and conservation actions. To address this gap, we suggest an increased emphasis on the application of existing knowledge to drive meaningful conservation outcomes and prioritization of new research on ongoing and emerging threats.

## Graphical abstract

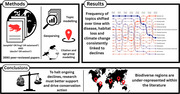

## Abstract (es)

Evolución de la investigación sobre la declinación mundial de anfibios

**Resumen:**

A finales de la década de 1980, la comunidad científica tomó conciencia de la grave y enigmática disminución de la población de anfibios. Esta disminución desencadenó una ola de investigaciones centradas en cuantificar su alcance e identificar los factores clave. Utilizamos técnicas de análisis de texto, como el modelado de temas y el análisis geográfico, para examinar la evolución de la investigación centrada en la disminución de los anfibios. Buscamos ofrecer un ejemplo de investigación científica en acción, desde el reconocimiento inicial y la cuantificación del fenómeno hasta la identificación de los factores y la comprensión de los mecanismos de la disminución de los anfibios. Delimitamos los temas de investigación, hicimos un seguimiento de las tendencias espaciotemporales desde 1985 hasta 2024 y obtuvimos el número de publicaciones por tema. Las primeras investigaciones se centraron en evaluar la veracidad de la disminución, a lo que siguieron investigaciones sobre los posibles factores determinantes (en particular, la radiación UVB, la contaminación y la fragmentación y pérdida de hábitats). Tras la identificación del hongo quitridio de los anfibios (*Batrachochytrium dendrobatidis*) a finales de la década de 1990, la investigación se centró en la enfermedad. Después de esto, la investigación centrada en las enfermedades se volvió cada vez más especializada, cuyos temas principales fueron la susceptibilidad, la resistencia y la tolerancia, y la mitigación. Más recientemente, el riesgo de extinción y el cambio climático se convirtieron en temas cada vez más destacados, lo que refleja las amenazas emergentes para los anfibios. Las regiones con alta biodiversidad de anfibios y disminuciones observadas (por ejemplo, América Central y del Sur) estaban infrarrepresentadas en la literatura, y la investigación se inclinaba fuertemente hacia América del Norte, Australia y Europa. Descubrimos una clara desconexión entre la literatura sobre la disminución de los anfibios y el desarrollo de medidas eficaces de gestión y conservación. Para cerrar esta brecha, sugerimos que se haga mayor hincapié en la aplicación de los conocimientos existentes para impulsar resultados significativos en materia de conservación y se dé prioridad a las nuevas investigaciones sobre las amenazas actuales y emergentes.

## 全球两栖动物数量下降的研究的演变

**【摘要】** 20世纪80年代末, 科学界开始意识到两栖动物正经历严重且难以理解的种群数量下降。这引发了一波研究热潮, 重点关注量化下降程度以及确定关键的驱动因素。本研究利用文本分析技术, 包括主题建模和地理解析, 分析了两栖动物数量下降研究的演变过程。我们试图通过案例来说明从最初认识和量化这一现象, 到确定驱动因素并了解两栖动物数量下降机制的科学探索过程。我们划分了研究主题, 追踪了1985‐2024年间的时空趋势, 并提取了每个主题的论文数量。我们发现, 早期研究的重点是评估两栖动物种群下降的真实性, 随后是调查潜在的驱动因素, 尤其是紫外线辐射、污染、栖息地破碎化和丧失。在20世纪90年代末发现蛙壶菌(*Batrachochytrium dendrobatidis*)后, 相关研究的重点转向了疾病。随后, 以疾病为重点的研究变得越来越特化, 其主题包括易感性、抵抗性和耐受性, 以及减缓影响。最近, 灭绝风险和气候变化成为日益突出的主题, 反映了两栖动物所面临的新兴威胁。两栖动物生物多样性高且已观察到数量下降的地区(如中美洲和南美洲)在文献中的代表性不足, 而研究更多地偏向于北美、澳大利亚和欧洲。我们还发现, 两栖动物数量下降的文献研究与有效管理和保护行动的发展之间存在明显的脱节。为了弥补这一差距, 我们建议应更重视应用现有知识来推动有意义的保护成果, 并优先考虑围绕当前存在威胁和新兴威胁开展新研究。**【翻译:胡怡思;审校:聂永刚】**

## INTRODUCTION

Amphibians are among the most imperiled vertebrate class globally; 41% of species are classified as threatened by the International Union for Conservation of Nature (IUCN) (Luedtke et al., 2023; Re:wild et al., 2023). Before 1980, long‐term amphibian population monitoring and understanding of population dynamics were limited, and the few documented declines were primarily attributed to habitat loss and related environmental factors (Cooke, 1972; Corn et al., 1989). Later reviews emphasized how early monitoring efforts were sporadic, lacked standard methods, and were often insufficient to detect long‐term trends (Alford & Richards, 1999; Houlahan et al., 2000). However, the 1980s marked a dramatic turning point. Reports of severe, enigmatic population declines and extirpations increased and left some scientists concerned (Alford & Richards, 1999; Pechmann & Wilbur, 1994).

The global amphibian decline phenomenon was acknowledged at the First World Congress of Herpetology in 1989 (Barinaga, 1990). Initially, there was debate about whether some of these declines were part of natural population fluctuations or driven by unknown factors (Alford & Richards, 1999; Blaustein & Wake, 1990; Carey et al., 1999; Pechmann & Wilbur, 1994). Although some population declines were linked to local drivers, such as land‐use change, many species disappeared from seemingly pristine, undisturbed habitats, leading to some declines being labeled “enigmatic” (Blaustein & Wake, 1990). Early reports documented local population losses (Blaustein & Wake, 1990; Crump et al., 1992), but accumulating data soon revealed that declines were occurring on a global scale, which led to the recognition of a “global amphibian decline phenomenon” (Alford & Richards, 1999; Berger et al., 1998). The severity and widespread nature of these losses underscored a broader biodiversity crisis, fueling fears of an impending sixth mass extinction (Beebee & Griffiths, 2005; Wake & Vredenburg, 2008).

Many amphibian declines were challenging to document due to their rapid onset and the often retrospective nature of investigations (Blaustein et al., 1994; Kuzmin, 1994; Pechmann & Wilbur, 1994; Sherman & Morton, 1993). Recognizing the need for a coordinated response, the IUCN launched the Declining Amphibian Populations Task Force (DAPTF) in the early 1990s to facilitate global monitoring and reporting (Vial & Saylor, 1993). Building on this, Blaustein et al. (1994) underscored that effective monitoring should span at least one generation of the species to distinguish genuine population declines from natural fluctuations. This highlighted 2 key challenges: the scarcity of long‐term monitoring efforts and the lack of standardized data collection methods. Researchers initially focused on resolving these challenges while investigating potential drivers of declines, which included UVB radiation, habitat loss, invasive species, chemical contamination, climate change, and disease (Alford & Richards, 1999; Barinaga, 1990; Blaustein et al., 1994; Pounds & Crump, 1994). Although these factors explained some localized losses, they could not account for many of the widespread declines reported globally (Blaustein et al., 2004; Carey & Alexander, 2003).

A breakthrough came in the late 1990s with the discovery of the amphibian chytrid fungus (*Batrachochytrium dendrobatidis*) (Bd), which was rapidly identified as a primary driver of many enigmatic declines, particularly in the Neotropics, Europe, the Americas, and Australia (Berger et al., 1998; Bosch et al., 2001; Carvalho et al., 2017; Grant et al., 2020; Longcore et al., 1999; Palomar et al., 2023; Scheele et al., 2019; Skerratt et al., 2007; Vredenburg et al., 2010; Whitfield et al., 2016). This discovery transformed amphibian research, igniting 3 major subdisciplines: amphibian disease ecology, disease epidemiology, and ecotoxicology. Researchers in these fields explored factors that included pathogen–host interactions, immune responses, environmental influences, and amphibian conservation with an emphasis on disease management (Bletz et al., 2024; Rosenblum et al., 2013). Research has included other pathogens, such as ranavirus (Blaustein et al., 2018; Brunner et al., 2021; Gray & Chinchar, 2015; Lesbarreres et al., 2011), parasites, and coinfections involving multiple pathogens (Herczeg et al., 2021). The identification of a second chytrid species, *Batrachochytrium salamandrivorans* (Bsal), in Europe in 2013 raised concerns about the potential for another amphibian pandemic; similar patterns of decline were observed in susceptible amphibian species (Bletz et al., 2024; Grant et al., 2015; Gray et al., 2023; Martel et al., 2013; Olson et al., 2024; Richgel et al., 2016; Yap et al., 2017).

The DAPTF initially focused on raising awareness, promoting research, and coordinating data collection on amphibian declines (Vial & Saylor, 1993). However, as understanding increased, a more action‐oriented approach was required. This led to the transition from DAPTF's information gathering role to the IUCN Species Survival Commission (SSC) Amphibian Specialist Group (ASG) in 2005, which brought together global experts to guide species‐specific conservation efforts. A pivotal step in this process was the launch of the first global amphibian assessment in 2004 (Stuart et al., 2004), which offered the first comprehensive evaluation of amphibian species worldwide. This assessment highlighted the global scale of the amphibian crisis and underscored the urgent need for coordinated conservation action. Since then, periodic updates, including the most recent global amphibian assessment (Luedtke et al., 2023; Re:wild et al., 2023) and the ASG's Amphibian Conservation Action Plan (Wren et al., 2024), have offered comprehensive overviews of species status, threats, and conservation strategies. These assessments revealed that habitat loss remains the leading threat and that climate change is emerging as a critical driver, potentially surpassing disease in significance (Bickford et al., 2024; Bletz et al., 2024; Luedtke et al., 2023; Wren et al., 2024). Regional trends have also shifted, with invasive species identified as a major threat in North America (Re:wild et al., 2023). Complementing these insights, Grant et al. (2023) provided a global perspective on amphibian conservation priorities, highlighting research gaps and aligning community perspectives with IUCN goals.

Four decades after the onset of the global amphibian decline phenomenon, there is the opportunity to examine how scientific inquiry has evolved in response to this pressing conservation issue. The rate and extent of these declines are unprecedented for any animal taxa, spatially and taxonomically (IPBES, 2019; Luedtke et al., 2023), prompting substantial global research efforts. Although significant progress has been made, amphibians remain understudied (González‐del‐Pliego et al., 2019; Grant et al., 2023; Womack et al., 2022). Understanding of amphibian diversity is still incomplete. Hundreds of new species have been described in recent decades, and the annual rate of new species descriptions remains high (AmphibiaWeb, 2025; Luedtke et al., 2023). Basic natural history data are still lacking for many species, and critical knowledge gaps persist in their ecology, behavior, and responses to emerging threats. Eleven percent (909 species globally) are still classified as data deficient, and the number of threatened species continues to rise (Luedtke et al., 2023). As major threats such as disease, habitat loss, invasive species, and climate change, intensify, evaluating past research efforts can help inform future conservation priorities.

Using text analysis techniques, we systematically tracked how research has evolved in response to the amphibian decline crisis from 1985 to 2024. We used this data‐driven approach to examine patterns and shifts in the research landscape that may not be immediately apparent through traditional literature reviews (Andrew et al., 2022; Evans et al., 2023; Westgate et al., 2015). We synthesized four decades of research to clarify how the scientific community has addressed global amphibian declines and to inform future conservation priorities. First, we compiled a comprehensive corpus of literature on amphibian declines in which we delineated 15 distinct research topics. We then examined spatiotemporal trends, topic interrelationships, and citation patterns to reveal shifts in research focus. Recognizing that different amphibian life stages face unique threats and require specific conservation strategies (Nolan et al., 2023), we also assessed the representation of different life stages within each research topic so as to identify potential knowledge gaps in amphibian biology and current conservation efforts.

## METHODS

### Search terms

We restricted our search to English‐language, peer‐reviewed literature on amphibian declines and/or extinctions. We did not include gray literature (texts published outside of scientific journals, such as reports, policy‐based literature, speeches, and news articles). We searched only for articles that had an abstract and were available online. This approach may underrepresent early studies or those published in print‐only journals; however, this standardized approach was necessary to facilitate text analyses.

In 2022, we searched 2 indexes, the Clarivate Analytics Web of Science Core Collection (WOS) and Scopus. We used the following Boolean search terms: (amphib* OR frog* OR salamand*) AND (declin* OR extinct*). The search field for WOS and Scopus was title, abstract, and keywords. The initial search yielded ∼13,000 articles, which we manually screened for duplicates and then further refined based on our inclusion criteria (outlined in “Inclusion criteria”). The corpus was updated on 17 February 2024 with the same search terms and search fields. We then used the same process to screen the corpus, with the exception that we used Covidence systematic review software (Covidence, 2024). All screening processes were based on titles and abstracts only (no full‐text screening was undertaken). Our final corpus contained 3590 articles (PRISMA diagram in Appendix S1).

Using the grep function in R studio 2023.9.1.494, we removed irrelevant characters or information (e.g., copyright information) from the titles and abstracts and excluded articles lacking abstracts to prepare the corpus for analysis.

### Inclusion criteria

All articles were reviewed by abstract and title only. We included articles in our corpus if they satisfied the following criteria. We selected articles published from 1 January 1985 to 17 February 2024 because this time frame effectively captures the development of research addressing the amphibian decline phenomenon, specifically the initial recognition of global amphibian declines and subsequent advancements in the field.

Publications were included if they focused on amphibians and addressed ecological or biological declines or extinctions in a contemporary context. Eligible studies included empirical data papers (e.g., field studies, population monitoring, ecological surveys); review articles with empirical data (e.g., meta‐analyses); taxonomic studies if they contributed to understanding species declines or extinction risks; and ecological laboratory‐based studies (e.g., experimental research on amphibians). We did not include human medical research on amphibians (e.g., studies in which amphibians were used as biomedical models unrelated to ecological or biological decline); paleontology studies (e.g., research on extinct amphibian species from prehistoric periods); general amphibian biology papers (e.g., studies on cellular function, genetics, or physiology unless directly related to a driver of decline, such as disease, habitat loss, or climate change); articles written in languages other than English; or gray literature (e.g., reports, policy documents, speeches, news articles, or other non‐peer‐reviewed manuscripts).

### Topic modeling

Topic modeling provides a data‐driven method for reviewing research (Roberts et al., 2014; Westgate et al., 2015). With this technique, topics are identified based on groups of words extracted from individual articles that frequently appear together, which allows each topic to represent a distinct and meaningful concept within the corpus. Each article is assumed to be composed of multiple topics, which allows the relative weight of each topic in a given article to be calculated. This analysis technique has been used widely across social sciences (Liu et al., 2016; Ramage et al., 2009; Westgate et al., 2015) but is comparatively rare in ecology and conservation, despite offering a way to effectively breakdown large corpora into thematic elements or topics (Westgate et al., 2015). Traditional review methods, such as meta‐analyses or systematic reviews, can be influenced by author bias, whether conscious or unconscious (Haddaway et al., 2020). Although topic modeling is not completely free from bias and limitations, it offers a more objective means of synthesizing large, complex bodies of research by focusing on patterns in language use (Westgate et al., 2015).

To prepare our corpus for topic modeling, we combined the titles and abstracts and then used the prepDocuments function in the stm package (Roberts et al., 2019) in R to reduce words to their root form (e.g., declin*for *declined*, *declining*, *declines*). We also removed stop words, numbers, and punctuation. Finally, we eliminated common and rare words, defined as those appearing in over 85% or fewer than 1% of articles, to prevent these words from disproportionately influencing topic identification (Westgate et al., 2015). This creates a representative sample of keywords for each article that was then used for analysis.

### Topics and topic titles

Using our prepared corpus, we applied structural topic modeling (STM), which we used to examine titles and abstracts of each article to assess the main topics of the corpus. We fitted our STM in the stm package with spectral initialization, a technique that efficiently captures the global structure of the data while ensuring stability and topic coherence (Roberts et al., 2016). To apply STM, the number of topics must be specified in advance. We tested a range of 10–25 topics before selecting 15, which we determined to provide a meaningful representation of the corpus without being too complex to interpret (Westgate et al., 2015). To aid in the interpretation of the topics, we gave each topic a summary title. Titles were assigned by 3 of the authors after reviewing the abstracts of the articles assigned to each topic and the 15 keywords provided as output from the STM associated with each topic (Andrew et al., 2022; Westgate et al., 2015) (Table 1).

**TABLE 1:** Fifteen topics identified from topic modeling of 3590 research articles on amphibian decline and associated keywords and topic descriptions.

Topic name^a^	Topic description	Top 15 weighted terms^b^
Amphibian declines	Global decline of amphibian populations	amphibian, popul, global, research, studi, caus, effect, impact, import, mani, ecolog, understand, can, human, provid
Population genetics	Genetic diversity within and among amphibian populations	popul, genet, divers, isol, gene, variat, structur, lineag, speci, genom, sequenc, two, rang, among, within
Disease ecology	Infection dynamics in amphibian populations, including host–pathogen interactions, susceptibility, and chytridiomycosis	infect, diseas, pathogen, host, dendrobatidi, chytridiomycosis, batrachochytrium, amphibian, popul, caus, fungus, emerg, chytrid, suscept, speci
Population status	Status of amphibian populations, including population distributions and trends	frog, site, popul, speci, survey, rana, rang, year, call, mountain, area, distribut, southern, observ, california
Climate change	Effects of climate on species distribution, specifically temperature, UVB radiation, and elevation effects	chang, climat, temperatur, environment, elev, increas, condit, speci, predict, amphibian, may, radiat, effect, uv‐b, rang
Monitoring methods	Models and methods for monitoring amphibian populations, including occupancy studies and sampling dynamics	popul, use, model, estim, detect, data, monitor, probabl, method, sampl, occup, studi, dynam, hellbend, trend
Breeding habitat	Breeding habitats of amphibians and the effects of landscape changes	habitat, pond, wetland, amphibian, landscap, area, speci, breed, use, urban, water, newt, road, land, agricultur
Parasites and environmental contaminants	Impact of environmental contaminants and parasites on amphibians	effect, exposur, amphibian, frog, parasit, treatment, increas, expos, surviv, develop, result, metamorphosi, larval, atrazin, reduc
Extinction risk	Assessment of the probability of species extinction based on population trends and threat exposure	speci, conserv, extinct, amphibian, distribut, threaten, area, assess, risk, threat, use, biodivers, reptil, region, current
Population dynamics	Impact of environmental and other factors on amphibian species abundance, diversity, and distribution, with a focus on salamanders	forest, salamand, abund, speci, stream, habitat, communiti, effect, amphibian, divers, rich, disturb, increas, signific, cover
Invasive species	Studies on the effects of invasive species like fish and bullfrogs on native amphibian populations	fish, nativ, predat, speci, invas, bullfrog, introduc, amphibian, presenc, food, may, introduct, effect, larva, experi
Chytridiomycosis	Quantification of *Batrachochytrium dendrobatidis* in amphibian populations, as well as the spread and impact of chytridiomycosis on amphibians	amphibian, dendrobatidi, batrachochytrium, sampl, pathogen, preval, detect, fungus, chytrid, speci, presenc, popul, bsal, collect, chytridiomycosi
Reproduction	Reproductive behaviors and success in amphibians	toad, breed, reproduct, popul, size, male, adult, surviv, femal, bodi, egg, bufo, individu, success, captiv
Tadpoles and environmental contaminants	Effects of contaminants, such as pesticides, on amphibian larvae	tadpol, effect, concentr, amphibian, pesticid, water, larva, toxic, expos, exposur, signific, develop, stage, studi, use
Skin function	Focused on bacterial communities, skin's defensive capabilities, and variation among species in resisting pathogens	skin, amphibian, communiti, immun, bacteri, function, frog, speci, bacteria, isol, inhibit, defens, pathogen, resist, differ

^a^ Names and descriptions were derived from the 15 most weighted terms associated with each topic.

^b^ The 15 most heavily weighted terms associated with each topic, as identified by the structural topic model. These include word stems generated during the text preprocessing stage (e.g., *divers* = diversity/diverse, *popul* = population/populations). These stems represent the most probable words associated with each topic, not exact keywords used in searches or indexing.

### Post Hoc Topic Model Analyses

Using the estimateEffect() function from the stm package in R, we examined changes in topic prevalence over time in our corpus. We treated year as a continuous predictor variable to examine its effect on the distribution of topic weights. This approach allowed us to consider the overall trajectory of each topic and the relative differences in prevalence over time. Consequently, we categorized topics as either hot (increasing in prevalence over time) or cold (decreasing in prevalence over time) (Westgate et al., 2015). We built a topic ranks model that incorporated 5‐year intervals as a factorial variable to ensure sufficient sample sizes while capturing meaningful research trends. We visualized these trends with the packages ggplot2 (Wickham, 2016) and ggbump (Sjoberg, 2020).

Topic modeling is based on the assumption that each article has some level of correlation with each topic (*n* = 15 in our study) in the corpus, ranging in magnitude from minimal (e.g., 0.02) to substantial (e.g., 0.98) (Westgate et al., 2015). Consequently, although some articles might have moderate associations with numerous topics, others may exhibit strong correlations with only a few topics (Westgate et al., 2015). To determine how each topic was distributed across the corpus, we calculated its average weight in unselected articles (those in which the topic was not the focus) and divided it by the average topic weight in selected articles (those in which the topic was the focus) (Westgate et al., 2015). This calculation provided a metric for which lower values indicated specific topics (low weight in unselected articles, high weight in selected articles) and higher values suggested more general topics (high weight in unselected articles, low weight in selected articles) (Westgate et al., 2015).

To examine the relationship between the prevalence of the 15 topics and how many times a paper was cited, we fitted a negative binomial generalized linear model (GLM) with the glmmTMB package (Brooks et al., 2017). Citation count was used as the response variable, logit‐transformed topic prevalence data for each topic were included as predictors, and years since publication accounted for the accumulation of citations over time. Given that our response was count data, we fitted the GLM with a negative binomial error distribution and a log link.

### Geographic entity extraction

We used the CLIFF‐CLAVIN geoparser in Python to identify geographic mentions in abstracts and titles (Evans et al., 2023; Gordon et al., 2023; Millard et al., 2020). This tool is designed to extract and identify geographic locations mentioned in text documents by performing named entity recognition to detect place names and then determine the primary geographic focus of the document, based on the locations’ frequency and prominence (D'ignazio et al., 2014). Because geographic mentions were identified solely from titles and abstracts, the locations primarily represented study sites, although other geographical references may also be captured during the process. We used a Docker container (Merkel, 2014) hosted on the GitHub repository (https://github.com/havlicek/CLIFF‐docker). CLIFF‐CLAVIN scans each article's title and abstract text for geographic place mentions, which we categorized into major country mentions and minor localities. We mapped these major mentions as country polygons, colored according to the number of unique mentions, and minor localities as points. We plotted the circular flags in Figure 4 with the ggflags package (Adams, 2024).

### Research focus across amphibian age classes

We examined the focus of amphibian decline research across different life stages (tadpoles, juveniles, and adults) because distinct age classes may experience unique threats and conservation challenges. By analyzing the representation of these life stages in each of the 15 topics, we aimed to identify broad trends and assess whether certain life stages are underrepresented, potentially indicating gaps in understanding of amphibian declines. To do this, we identified articles containing the key terms tadpol*, larva*, juvenil*, metamorph*, and adult* and a grouped category for studies addressing multiple age categories (mention of more than one key term [tadpole* and/or adult*, and/or juvenil*). For clarity in analysis, the categories were further consolidated. *Tadpole* represented both *tadpole* and *larvae* variations, and *juvenile* encompassed *metamorph* and *juvenile*; *adult* remained as a distinct category. Although this approach provides a valuable large‐scale overview of age classes studied within the corpus, our reliance on abstract text for this analysis did not capture all mentions of age classes present in the full text.

## RESULTS

### Topics

We delineated 15 topics from our corpus of 3590 research articles (Table 1). The topics represented a range of key research themes, such as population dynamics, disease ecology and prevalence, environmental contaminants, and climate‐related research. Table 1 provides descriptions and keywords from the STM output.

### Topic prevalence over time

**FIGURE 1:**
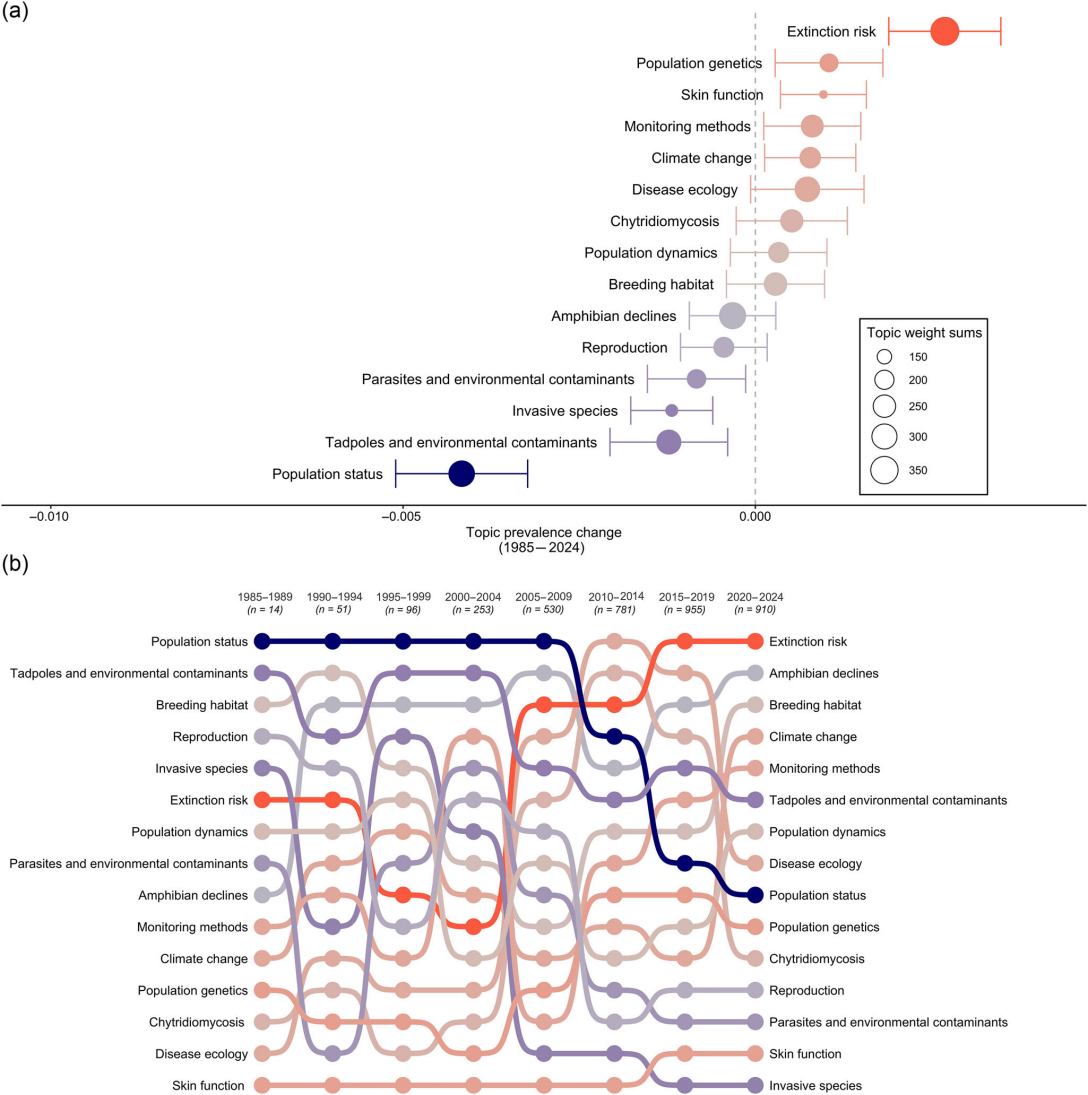
Relative to the amphibian decline literature from 1985 to 2024, changes in topic (a) prevalence (topics to the right of the dashed line, increase; topic to the left of the dashed line, decrease; circle size, proportional to total topic weight and indicates the cumulative contribution of a topic across all documents in the corpus; error bars, 95% confidence intervals) and (b) focus of research (topics ranked based on prominence in the literature for each half decade; topic colors correspond to topic prevalence results in [a]).

Our analyses revealed major changes in topic prevalence over time. Extinction risk was the most rapidly growing topic, whereas population status, once a dominant focus, declined significantly (Figure 1a). Extinction risk had the largest total topic weight (364), followed by amphibian declines (322). Despite its decline in prevalence, population status was the third largest topic (total topic weight 311). Examining changes across 5‐year intervals revealed shifts in research focus over time (Figure 1b). From 1985 to 1994, research primarily centered on tadpoles, environmental contaminants, and broader topics, such as breeding habitat and reproduction. From the early 2000s onward, there was a noticeable shift toward disease‐related topics, including parasites, environmental contaminants, disease ecology, and studies on chytridiomycosis, reflecting advances in understanding amphibian diseases. From 2010 to 2019, there was a surge in climate change research, highlighting the growing recognition of its global impact. Interest in some topics, such as breeding habitat and reproduction, fluctuated, whereas other topics, such as invasive species, steadily declined. Overall, our findings revealed a transition from broader population studies to a more detailed exploration of the factors threatening amphibians, particularly extinction risk, disease, and climate change.

### Topic specificity and generality

**FIGURE 2:**
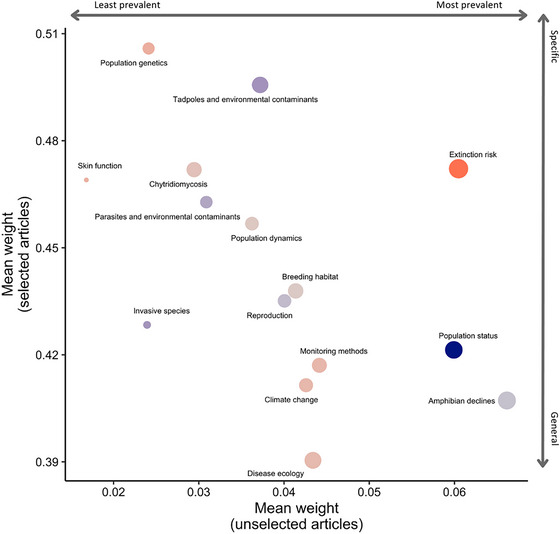
Specificity and generality of research topics in the amphibian decline literature (*x*‐axis, mean topic weight in unselected articles [articles in which the topic was present but not dominant]; *y*‐axis, mean topic weight in selected articles [articles in which the topic was the highest probability topic, following Westgate et al. {2015}]; circle size, proportional to the topic's overall prevalence across the corpus; color shading, relative topic prevalence from Figure 1; dark blue, least prevalent; orange, most prevalent).

General topics included amphibian declines, population status, disease ecology, climate change, and monitoring methods. More specific topics included population genetics, tadpoles and environmental contaminants, skin function, and chytridiomycosis (Figure 2). Skin function was the least prevalent topic in the corpus, and amphibian declines was the most prevalent (Figure 2).

### Influence of citations on topic ranking

**FIGURE 3:**
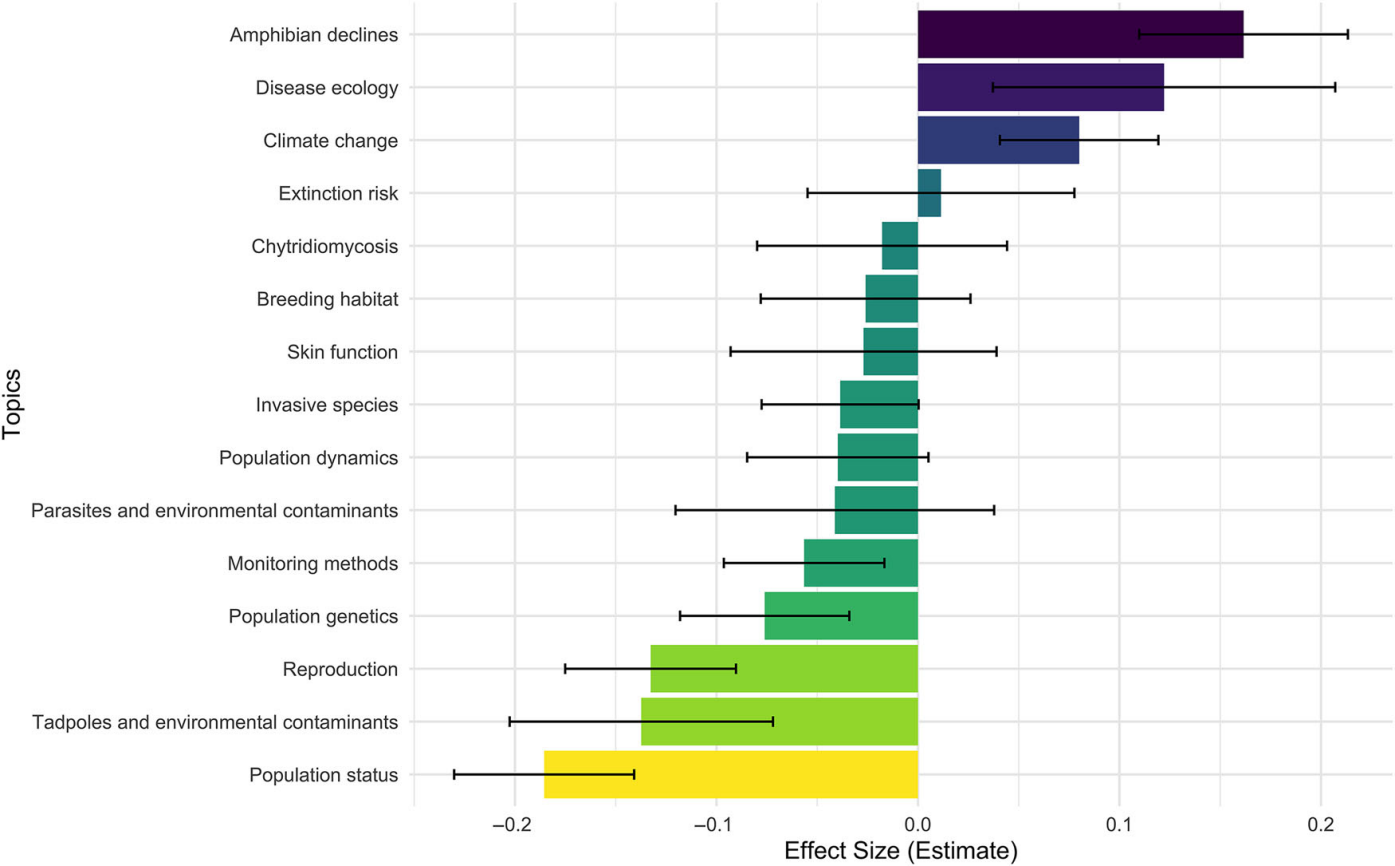
Estimated change in citation counts of articles on amphibian decline associated with a 1 SD increase in the prevalence (transformed to the logit) of the topic (error bars, 95% confidence intervals).

We found significant positive and negative associations between topic prevalence and citation counts (Figure 3; Appendix S2). Some topics, such as amphibian declines (estimate = 0.162, *p* < 0.001), disease ecology (estimate = 0.122, *p* < 0.001), and climate change (estimate = 0.079, *p* < 0.001), exhibited significant positive effects, indicating that higher prevalence of these topics in a paper was associated with increased citation counts (Figure 3). Conversely, other topics, such as population genetics (estimate = −0.076, *p* < 0.001), population status (estimate = −0.186, *p* < 0.001), monitoring methods (estimate = −0.057, *p* < 0.001), reproduction (estimate = −0.133, *p* < 0.001), and tadpoles and environmental contaminants (estimate = −0.137, *p* < 0.001), were negatively associated with citation counts, suggesting that papers focused more heavily on these topics received fewer citations (Figure 3). Some topics, such as breeding habitat, parasites, environmental contaminants, extinction risk, chytridiomycosis, and skin function, did not have statistically significant associations, indicating that the prevalence of these topics did not significantly influence citation counts. Finally, years since publication (estimate = 1.097, *p* < 0.001) was positively associated with citation counts, meaning that older papers tended to receive more citations (Appendix S2).

### Geoparsing

**FIGURE 4:**
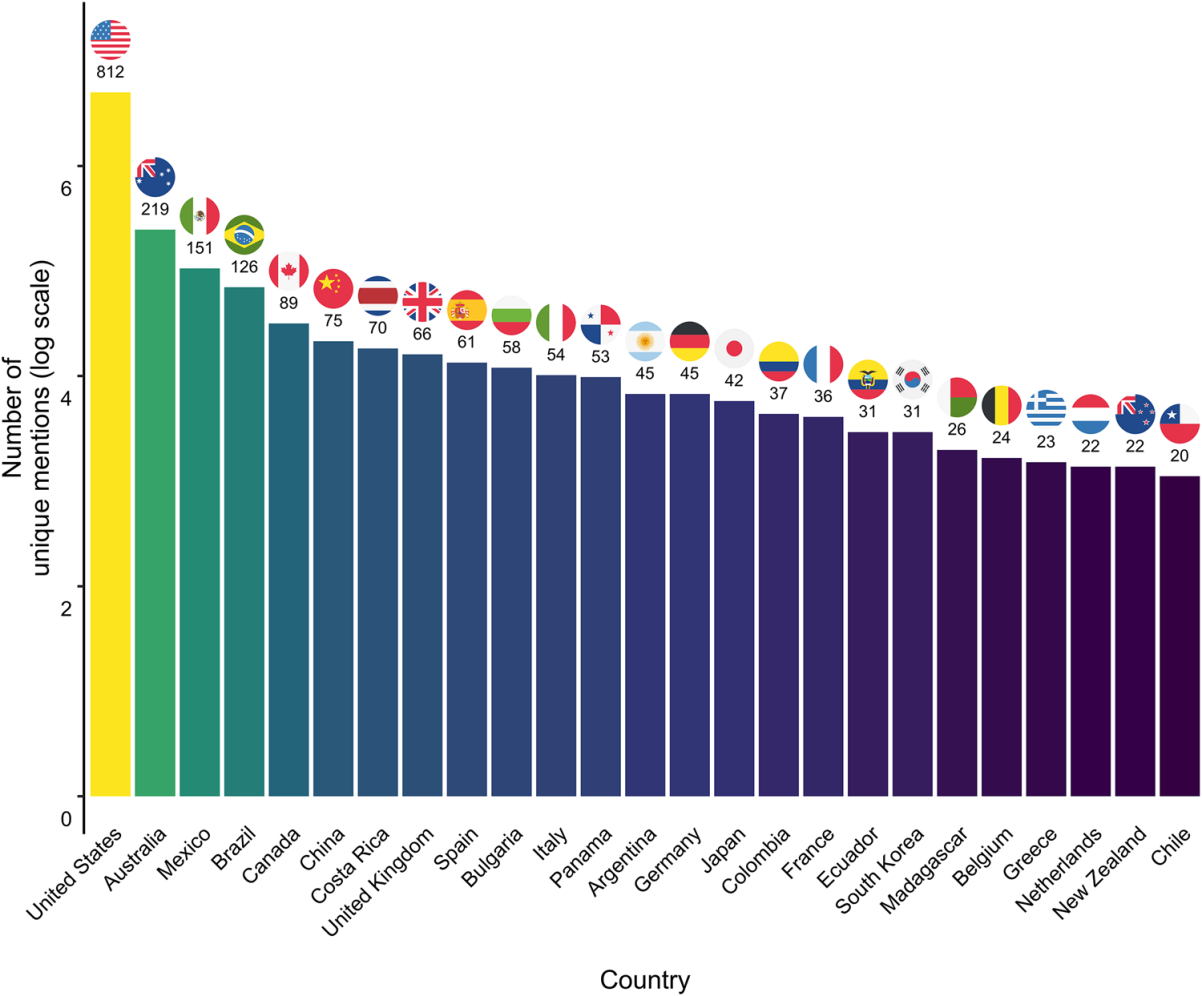
Twenty‐five countries with the most unique mentions in the amphibian decline corpus (numbers above bars, number of unique mentions per article).

The United States had the largest number of geographic mentions in our corpus, meaning the United States was mentioned in the title or abstract of the paper, followed by Australia and then Mexico. Overall, 137 countries were mentioned in the corpus, and the top 25 are visualized in Figure 4.

**FIGURE 5:**
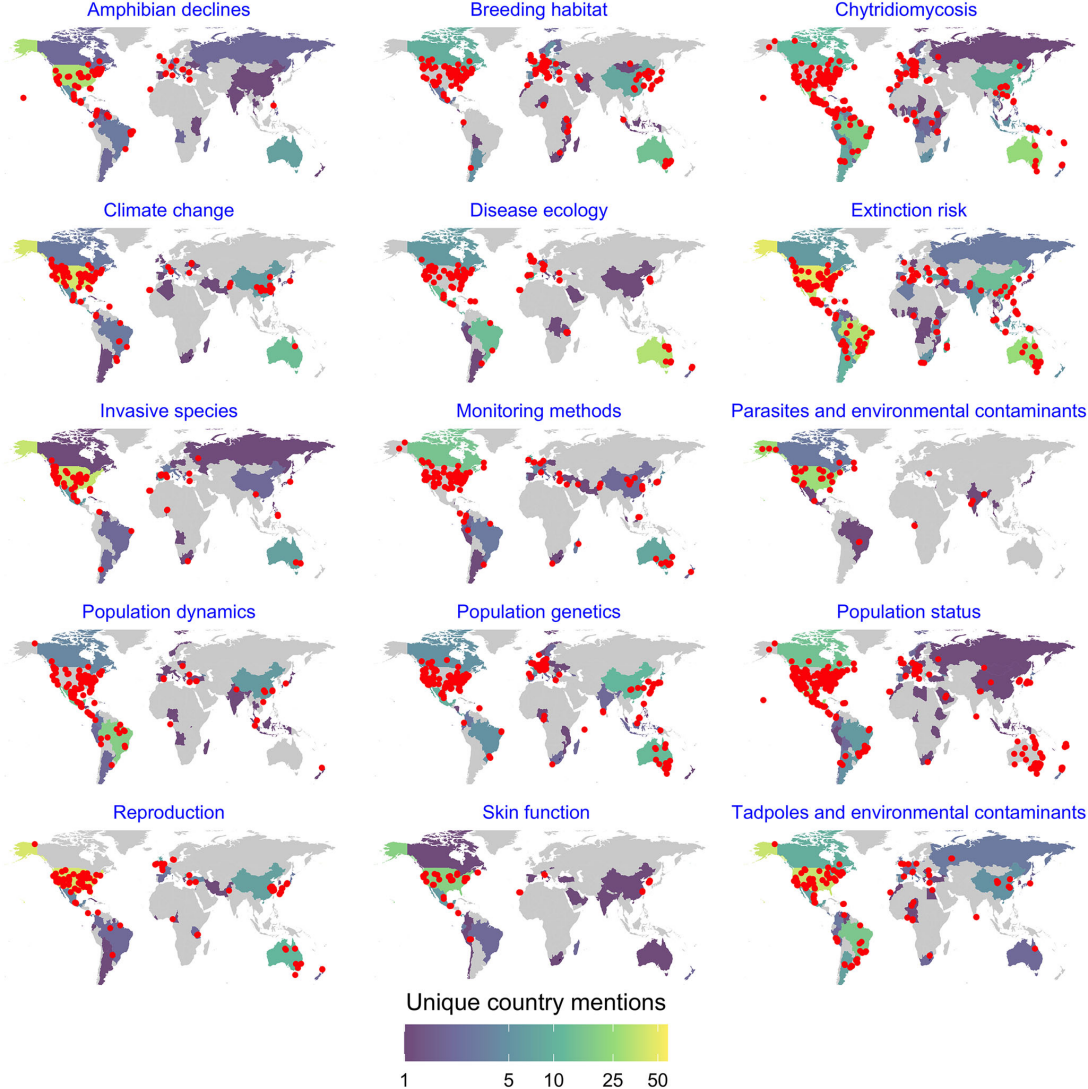
Geographic distribution of 15 research topics in the corpus of the amphibian decline literature (shading, mentions per article; yellow, ≥50 mentions; red points, specific locations mentioned in articles).

**FIGURE 6:**
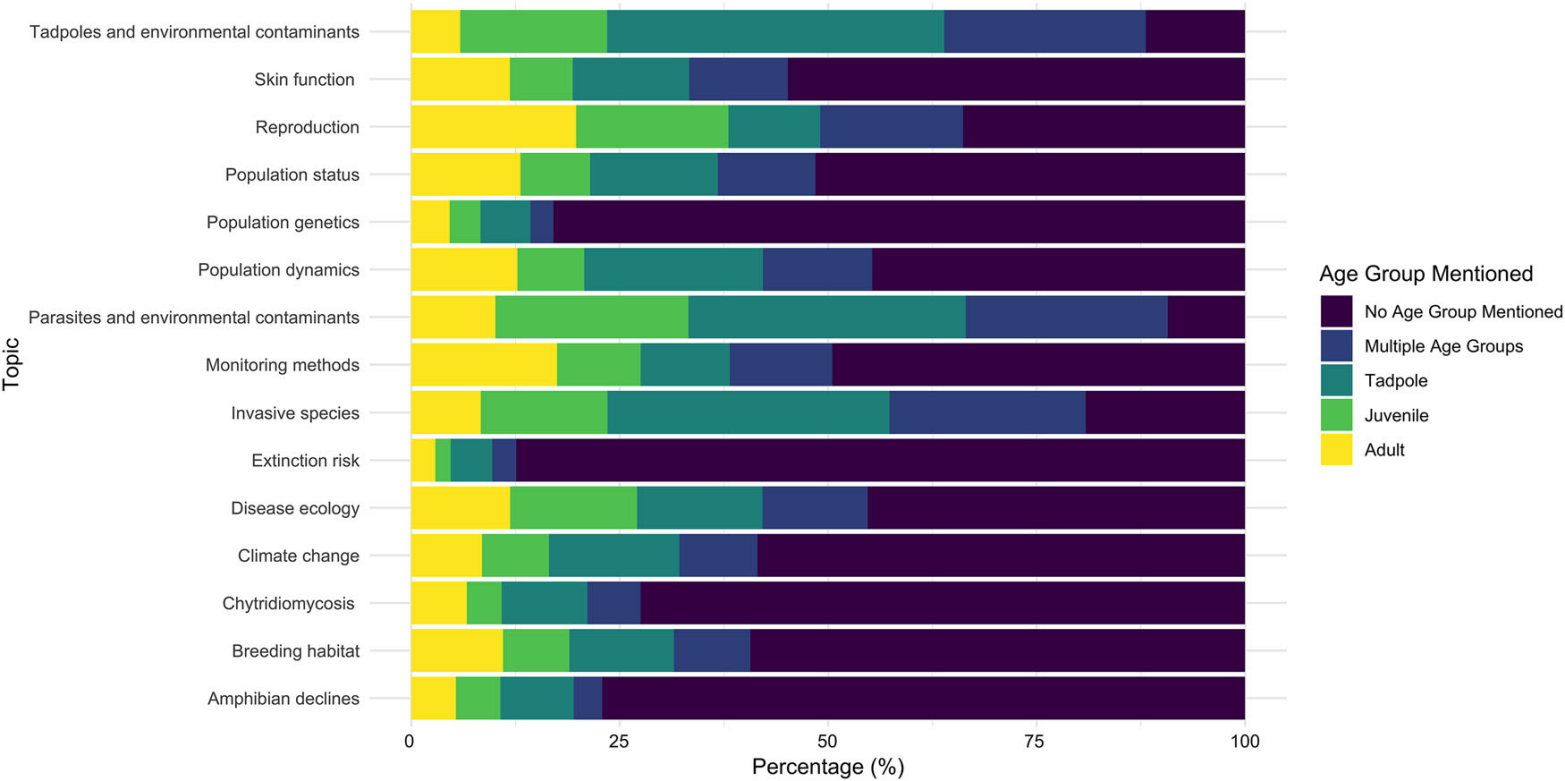
Percentage of articles mentioning different amphibian age groups (adult, juvenile, tadpole, combination of multiple age groups, or no age group mentioned) for each of the 15 identified research topics in the amphibian decline research literature (1985–2024) (juvenile includes juvenile and metamorph; tadpole includes tadpole and larvae; dark purple, no age group specified).

The geographic distribution of research topics in the corpus showed that many studies were concentrated in North America, Europe, and Australia (Figures 4 & 5). Certain topics, such as chytridiomycosis and disease ecology, were particularly prevalent in the Americas (Figure 5). Similarly, some topics, such as population genetics and monitoring methods, were heavily focused on North America and Europe. Although Figures 5 and 6 indicate some level of global coverage, Africa, Southeast Asia, and South America were notably underrepresented in comparison. The specific locations mentioned in the articles (denoted by red dots) largely aligned with the broader regions associated with each topic. These mentioned locations were largely concentrated in biodiversity hotspots or areas where amphibians face major threats. Because geographic mentions were identified solely from titles and abstracts, the locations presented primarily represented study sites, although it must be noted that other geographical references also may have been captured during the extraction process.

### Age class focus across research topics

The age groups of amphibians studied varied among topics. Every topic included mentions of all 3 age groups: adult, juvenile, and tadpole. Some topics, such as tadpoles and environmental contaminants and invasive species, had a high proportion of articles focused on tadpoles, whereas reproduction and monitoring methods had a higher percentage of articles focused on adults. Juveniles were the least mentioned across all topics.

## DISCUSSION

### Tracking the scientific community's exploration of the amphibian decline phenomenon

Our results traced the evolution of research on amphibian declines. Early studies focused on quantifying population declines and establishing baseline population dynamics. As the severity of declines became evident, attention turned to identifying potential drivers, especially those that may have accounted for enigmatic declines that were not associated with overt habitat changes. These included UVB radiation, invasive species, and pollutants (Barinaga, 1990; Blaustein et al., 1994; Herman & Scott, 1992). This trend was evident in the results from our topic model, where research on environmental contaminants, parasites, and climate change was particularly prominent from 1990 to 2005 (Figure 1). Some hypothesized drivers, such as UVB radiation, were later deemed less influential (Licht, 2003), whereas others, for example, invasive species, gained recognition as major threats (Falaschi et al., 2020) and shaped research focus for subsequent years (1995–1999) (Figure 1).

The discovery of Bd in the late 1990s (Berger et al., 1998; Longcore et al., 1999) and its catastrophic impact on amphibian populations triggered a surge in disease research (Carvalho et al., 2017; Hero & Morrison, 2004; Lips, 1999; Muths et al., 2003; Vredenburg et al., 2010). This led to a global effort to map the spatiotemporal spread of Bd and its ecological impacts (Fisher et al., 2009; Olson et al., 2013, 2021; Scheele et al., 2019; Skerratt et al., 2007). From 2000 onward, disease‐related topics dominated our topic rankings. Disease ecology and chytridiomycosis were consistently ranked at the top alongside extinction risk and amphibian decline (Figure 1). The identification of Bsal in 2013 (Martel et al., 2013) and its large negative effects on European salamanders intensified concerns about a new wave of declines, particularly if the pathogen were to enter North America, which has the highest salamander diversity worldwide (Gray et al., 2023; Grear et al., 2021; Richgels et al., 2016; Yap et al., 2017). This concern was evident in our topic model, with renewed emphasis on population dynamics after 2000 and again after 2015 (Figure 1). Other foundational topics, such as monitoring methods, breeding habitat (potentially representing a renewed focus on habitat loss), and climate change, also increased in the last decade (Figure 1). Although population genetics emerged as a distinct topic, key concepts from landscape genetics, such as gene flow and connectivity, did not appear among the most prominent terms (Table 1). This likely reflected their relatively low frequency in titles and abstracts or their distribution across multiple topics, rather than a lack of relevance to amphibian conservation. Lastly, extinction risk remained the top‐ranked topic in our topic model (Figure 1), underscoring the enduring severity of amphibian declines. Our findings align with broader literature indicating that amphibian declines remain largely unmitigated and that extinction risks continue to escalate in response to emerging threats (Figure 1; Grant et al., 2023, 2019; Luedtke et al., 2023). As the drivers of decline continue to evolve, so too must the scientific priorities so that research efforts are responsive to the complex and shifting challenges amphibians face.

### Translating research into conservation action

Our analyses revealed a limited focus on conservation and management in the corpus. No explicit management‐based topics emerged, and management‐associated terms were largely absent from the keywords for each topic (Figure 2; Table 1). This, in part, could be an artifact of our search terms, which focused on amphibian declines and extinctions. However, other authors argue that conservation remains a comparatively underrepresented topic in amphibian decline research (Grant et al., 2023, 2019). More broadly, there is a notable disconnect between scientific knowledge and action in conservation as a whole (Cook et al., 2013; Grant et al., 2019; Toomey et al., 2017). Our analyses highlighted that although conservation strategies are often informed by a diverse range of research topics, this connection is not always clear in the literature. For example, several of our identified topics included keywords related to the impacts of invasive fish (Table 1), which directly inform management strategies, such as fish removal, to restore amphibian habitats (Vredenburg, 2004). However, the link between research findings and practical conservation actions is often not formally documented, making it difficult to trace how scientific knowledge is translated into management practices. To bridge this gap, we advocate for a more intentional approach in which researchers explicitly document and publish the conservation actions that could and should arise from their findings. This should encompass not only direct management actions (e.g., habitat restoration, reintroductions, captive rescue colonies, invasive species removal) but also the foundational research that underpins those actions (e.g., genetic studies to guide translocations, disease risk assessments, habitat suitability modeling). By making these connections both visible and actionable, we can stimulate idea generation and more effectively bridge the gap between knowledge and action.

Another contributing factor may be that conservation‐oriented research is often published outside peer‐reviewed scientific journals, commonly appearing in government reports, conservation action plans, and gray literature, which are not typically indexed in academic search engines. For example, federal regulations in the United States prohibit the importation of salamanders to prevent the introduction of Bsal (U.S. Fish & Wildlife Service, 2025), and similar protective rules have been implemented in Canada (Olson et al., 2024). Incorporating nonscientific literature could provide a more comprehensive view and should be considered in future analyses. However, this may also be an indication of limited success in bridging the gap between research and action. Current habitat protection efforts for amphibians are providing insufficient scale to effectively reduce extinction risk (Grant et al., 2023; Luedtke et al., 2023; Nori et al., 2015). Similarly, practical mitigation strategies for Bd and Bsal remain largely ineffective in natural ecosystems (Garner et al., 2016). Grant et al. (2023) similarly conducted a text analysis in which they used *amphibian conservation* as a search term to extract topics from five key conservation journals over 5 years (2015–2020). These data were compared to identified IUCN research priorities and a community consensus obtained from surveying the herpetological research community directly. Despite targeting conservation‐specific literature, they found that research is not always effectively translated into conservation outcomes and that current research aims in the literature were not aligned with the key aims of the research community and IUCN‐identified priorities (Grant et al., 2023). Grant et al. (2023) also identified 3 underrepresented but critical research areas: genetics and genomics, climate change impacts, and human dimensions of conservation. Similar trends are reflected in our results. Population genetics rose in rank over the past decade but remained comparatively low, despite conservation genetics being identified in the 1980s as a primary need in the nascent discipline of conservation biology (see Meine et al., 2006). Overall, very few recognizably human‐oriented topics (besides environmental contaminants) emerged from the corpus (Figure 1).

The next challenge in conservation research is the significant investment of time, human resources, and funding required for applied conservation efforts (Walls, 2018). In contrast, academia tends to prioritize highly citable work in high‐impact journals, which often favors broad theoretical contributions over applied, region‐specific research (Fischer et al., 2012). This misalignment of priorities has been highlighted by Choi et al. (2024), who argue that there is a fundamental mismatch between incentives for high‐impact publication and the practical implementation of conservation science. They also emphasize the role of low‐impact‐factor journals in disseminating case studies and applied research critical for conservation success, which often receive less recognition despite their practical value (Choi et al., 2024). Our results reflected this trend. Generalized, globally relevant topics ranked higher than region‐specific or specialized research (Figures 1 & 2). The prevalence of basic monitoring topics, such as population status, greatly declined over time (Figure 1). This decline may reflect either a shift in the meaning of this topic over time or a reduced emphasis on direct population monitoring itself. This shift in focus may stem from the same systemic challenges, with long‐term population monitoring likely not considered novel enough to attract funding or publication in high‐impact journals (Choi et al., 2024; Grant et al., 2023). As a result, critical data on amphibian population dynamics, a cornerstone of decline research, may be increasingly overlooked and lead to gaps in the ability to track and understand long‐term trends. Expanding community science initiatives may offer a valuable strategy for bridging this gap. Public participation in data collection, disease surveillance, and habitat monitoring has already proven successful in various research and conservation efforts (Lawson et al., 2015; Petrovan & Schmidt, 2019; Rowley et al., 2024). Improving public engagement and integrating local knowledge could help elevate amphibian declines in funding and policy priorities (Olson & Pilliod, 2022). Additionally, by broadening the network of contributors to science, community‐driven efforts could provide locally relevant data and long‐term monitoring that are currently underrepresented in peer‐reviewed research.

Although data‐driven conservation remains foundational, it is important to recognize that waiting for complete certainty can delay essential actions (Brunner et al., 2021; Scheele et al., 2018). To close the gap between research and conservation actions, we emphasize the need to strike a balance between caution and proactive intervention. Embracing calculated risk taking is essential to progress the development of effective management actions (Scheele et al., 2018). Proactive management, such as initiating conservation actions before significant declines are observed, can be more effective and cost‐efficient than reactive measures. For instance, Moor et al. (2022) demonstrated that large‐scale pond creation in Switzerland led to substantial recovery of multiple amphibian species, highlighting the benefits of general habitat restoration. Similarly, adaptive management strategies, including assisted colonization, have shown promise for the critically endangered northern corroboree frog (Scheele et al., 2022). More broadly, conservation translocations for species threatened by chytrid fungus hold considerable potential. Scheele et al. (2021) provide a structured framework for assessing disease risks and habitat suitability before release. These examples underscore that integrating evidence‐based risk taking into conservation planning is crucial for effective amphibian conservation.

### Geographic biases in the literature

Geographical regions with high biodiversity and ongoing declines remain underrepresented in the literature (González‐del‐Pliego et al., 2019; Grant et al., 2023). We found a strong geographic bias toward research conducted in North America, Europe, and Australia (Figures 5 & 6). In contrast, amphibian populations in other areas, such as the Caribbean islands, Madagascar, South America, and Southeast Asia, have undergone inferred population declines since the outset of the global amphibian decline crisis (Luedtke et al., 2023; Scheele et al., 2019; Stuart et al., 2004) yet are notably underrepresented in our geoparsing (Figure 4). This bias extended beyond geographic representation to long‐term monitoring efforts. We found that research on population status, population dynamics, monitoring methods, and amphibian decline was less prevalent in these high‐priority regions, despite ongoing threats, such as habitat loss and climate change (Luedtke et al., 2023). The severity of this underrepresentation is highlighted in Figure 5, where many of these same regions appeared prominently within the extinction risk topic, suggesting that although species in these areas are at high risk, research efforts remain limited. Without substantial research efforts in the regions most affected by amphibian declines, understanding will remain incomplete, and effective intervention strategies will continue to fall short.

Addressing this geographical bias should be a research priority to support meaningful, globally relevant conservation actions capable of halting the ongoing amphibian decline crisis. However, assessing declines on a global scale presents challenges, particularly in high‐biodiversity regions where monitoring is limited or fragmented (Howard & Bickford, 2014; Nori et al., 2018). Insufficient research efforts likely result in undetected declines, suggesting that the actual level of threat in these regions may be greater than perceived (Hof et al., 2011; Grant et al., 2023). Addressing these knowledge gaps requires comprehensive global data on research efforts and sampling intensity and greater collaboration with local researchers, conservation practitioners, and stakeholders (Arlettaz et al., 2010; Grant et al., 2019). However, these efforts are compounded by broader systemic barriers, such as unequal access to funding, conflicting political priorities, and land‐use pressures (Adenle et al., 2015; Grant et al., 2023, 2019; Rands et al., 2010). The dominance of English in academic publishing (Amano et al., 2016) poses a further challenge. Use of only English‐language articles, as in our study, excludes critical research published in other languages. However, there are difficulties in running and comparing multilingual text analysis models (Nicholas & Bhatia, 2023). Tackling a global crisis, such as amphibian decline, requires more inclusive research approaches, including greater representation of non‐English‐language studies and multilingual collaboration. Addressing these disparities will be essential for building a more comprehensive and actionable understanding of amphibian declines worldwide.

### Research focus across amphibian life stages

All amphibian life stages were observed across all research topics in various proportions (Figure 6). Logically, tadpoles were the most mentioned in the topics directly related to their age class, such as tadpoles and environmental contaminants. However, we expected to see a higher proportion of tadpoles mentioned relative to the topic of reproduction (Figure 6). A significant proportion of articles across all topics did not explicitly mention any of the life stages in their titles or abstracts. This lack of data on amphibian life stages could hinder the ability to consider age‐specific risks for amphibians, such as disproportional mortality of juveniles on roads during dispersal (Petrovan & Schmidt, 2019). However, our use of abstracts rather than full texts may have contributed to some degree of underrepresentation in our data.

### Citations and topic prevalence

Several topics, such as amphibian declines, disease ecology, and climate change, showed a positive association with citation counts, suggesting that papers within these topics received more attention during the research period 1985–2024. This aligns with the prevalence results we observed in our topic analysis; these topics showed some of the highest increases in prevalence over time (Figure 1) and were among the most general (defined by the generality score from our topic model) topics in the corpus (Figure 2). General topics with broader appeal and therefore broader audiences are more likely to accrue citations over time compared to specialized topics. Consequently, the trajectory of amphibian decline research may appear skewed toward these general themes, potentially overshadowing advances in narrower yet equally important research areas.

Specialized topics (again, defined by the generality score), such as population genetics and reproduction, were negatively associated with citation counts, implying that papers focused on these areas attracted a narrower audience. Of particular interest is the strong negative association between population status and citation counts. Although population status was general, heavily weighted, and consistently prevalent from 1985 to 2009 (Figures 4 & 5), its lower citation rate may again reflect a shift in research focus from foundational monitoring of declines to identifying and addressing specific drivers. Alternatively, this pattern may suggest that although population status remained an essential component of amphibian decline research, it was less frequently cited outside of its immediate subfield. The declining citation rates for such studies may reflect a shift in focus of the field, where baseline assessments are less frequently cited in favor of other research, and a differential recognition of research across subfields.

We quantified four decades of amphibian decline research and found a clear evolution from verifying declines to determining their drivers and exploring mitigation strategies. Nonetheless, conservation and management are underrepresented in the literature, reflecting a broader gap between research and practical implementation (González‐del‐Pliego et al., 2019; Grant et al., 2023; Womack et al., 2022). Our findings further highlight this disconnect between scientific knowledge and conservation action. By quantifying research trends, our study complements recent global syntheses on species status and threats (Grant et al., 2023; Luedtke et al., 2023; Wren et al., 2024) and echoes the significant geographic gaps in research that have been identified, leaving many biodiversity hotspots and amphibian populations understudied.

As amphibians face accelerating threats from habitat destruction, climate change, and disease (Luedtke et al., 2023; Wren et al., 2024), targeted action is critical. However, conservation efforts are often hindered by a lack of comprehensive data, which can lead to hesitation in decision‐making. We advocate for proactive conservation strategies that incorporate calculated risk taking, acting on the best available evidence while remaining responsive to new information. Prioritizing scalable strategies, such as habitat restoration, is critical. Balancing data‐driven decisions with the flexibility to act under uncertainty may ensure that amphibian conservation efforts are evidence based and adaptive.

As biodiversity faces an increasing array of threats, our data‐driven evaluation of scientific inquiry in action provides several broader lessons applicable to researchers tackling parallel conservation challenges. First, establishing baseline data on species’ ecology, distributions, and abundance is crucial for accurately assessing population trends and identifying notable declines. Second, in an era of unprecedented global change, researchers must remain open to unexpected drivers of species decline, ensuring that conservation research is adaptive and responsive. Finally, although identifying declines and their causes is vital, these steps are just the beginning. Effective conservation requires proactive management and sustained efforts to halt the erosion of Earth's biological diversity.

## Supporting information



Supporting Appendices

Supporting Information
