# Supplementary material for: Evolution of research on global amphibian declines
Source: Conserv Biol. 2025 Sep 12;40(1):e70146. doi: 10.1111/cobi.70146 (PMC12856797; doi:10.1111/cobi.70146)
Supplement: Supplementary file 1 — Supporting Appendices [file COBI-40-e70146-s002.docx]

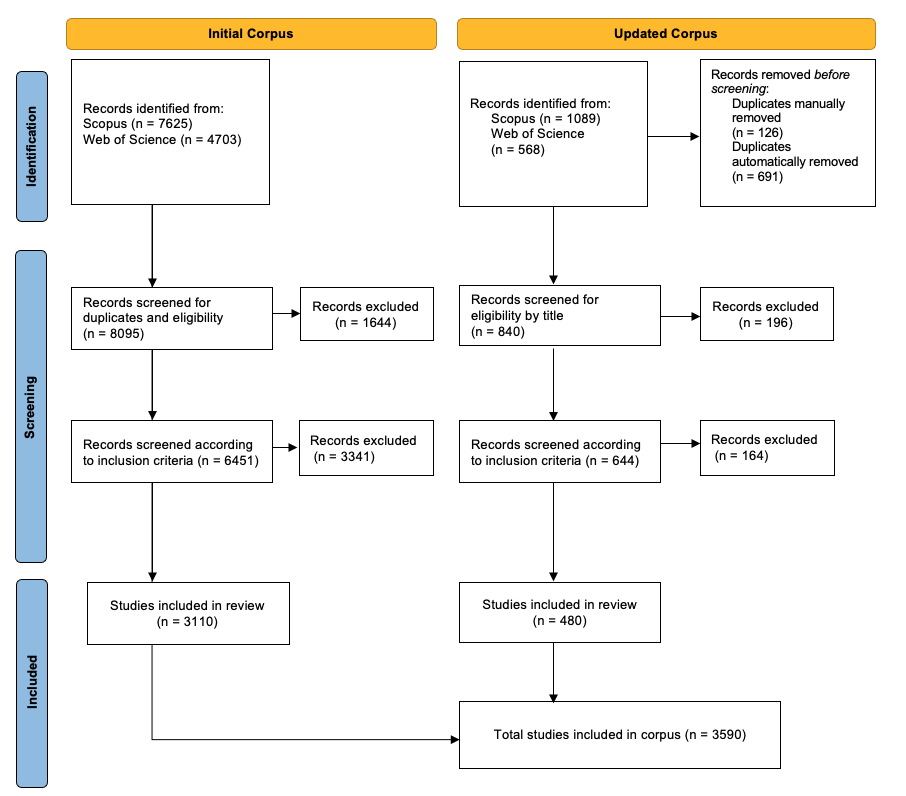


**Appendix S1.** PRISMA diagram outlining the review process for both the initial and the updated corpus.

**Appendix S2** Effect sizes of 15 topics on citation counts using a negative binomial regression model. The table shows the estimates, standard errors, z-values, and p-values for each topic, as well as the effect of years since publication. Statistically significant effects (p < 0.05) are bolded.

| **Predictor variables** | **Estimate** | **Std Error** | **z** | **p** |
| --- | --- | --- | --- | --- |
| *Intercept* | 3.336 | 0.017 | 195.935 | <0.001 |
| **Amphibian declines** | **0.162** | **0.026** | **6.120** | **0.000** |
| **Population genetics** | **-0.076** | **0.021** | **-3.549** | **0.000** |
| **Disease ecology** | **0.122** | **0.043** | **2.818** | **0.005** |
| **Population status** | **-0.186** | **0.023** | **-8.140** | **0.000** |
| **Climate change** | **0.080** | **0.020** | **3.987** | **0.000** |
| **Monitoring methods** | **-0.056** | **0.020** | **-2.779** | **0.005** |
| Breeding habitat | -0.026 | 0.027 | -0.978 | 0.328 |
| Parasites and environmental contaminants | -0.041 | 0.040 | -1.023 | 0.306 |
| Extinction risk | 0.011 | 0.034 | 0.338 | 0.735 |
| Population dynamics | -0.040 | 0.023 | -1.733 | 0.083 |
| Invasive species | -0.039 | 0.020 | -1.944 | 0.052 |
| Chytridiomycosis | -0.018 | 0.032 | -0.563 | 0.574 |
| **Reproduction** | **-0.133** | **0.022** | **-6.135** | **0.000** |
| **Tadpoles and environmental contaminants** | **-0.137** | **0.033** | **-4.119** | **0.000** |
| Skin function | -0.027 | 0.034 | -0.802 | 0.423 |
| **Years since publication** | **1.097** | **0.018** | **59.744** | **<0.001** |
